# Clinical Impact of Preoperative Relief of Jaundice Following Endoscopic Retrograde Cholangiopancreatography on Determining Optimal Timing of Laparoscopic Cholecystectomy in Patients with Cholangitis

**DOI:** 10.3390/jcm10194297

**Published:** 2021-09-22

**Authors:** Kyu-Hyun Paik, Yoon Suk Lee, Won-Suk Park, Yong Chan Shin, Woo Hyun Paik

**Affiliations:** 1 Department of Internal Medicine, St. Mary’s Daejeon Hospital, College of Medicine, The Catholic University of Korea, Daejeon 34943, Korea; qhyun515@naver.com (K.-H.P.); mdonekr@naver.com (W.-S.P.); 2 Department of Internal Medicine, Inje University Ilsan Paik Hospital, Goyang 10380, Korea; lys0326@hanmail.net; 3 Department of Surgery, Inje University Ilsan Paik Hospital, Goyang 10380, Korea; ycshindr@gmail.com; 4 Department of Internal Medicine, Liver Research Institute, Seoul National University Hospital, Seoul National University College of Medicine, Seoul 03080, Korea

**Keywords:** ERCP, cholecystolithiasis, choledocholithiasis, laparoscopic cholecystectomy, cholangitis

## Abstract

Background: About 10% of patients with gallbladder (GB) stones also have concurrent common bile duct (CBD) stones. Laparoscopic cholecystectomy (LC) after removal of CBD stones using endoscopic retrograde cholangiopancreatography (ERCP) is the most widely used method for treating coexisting gallbladder and common bile duct stones. We evaluated the optimal timing of LC after ERCP according to clinical factors, focusing on preoperative relief of jaundice. Methods: A total of 281 patients who underwent elective LC after ERCP because of choledocholithiasis and cholecystolithiasis from January 2010 to April 2018 were retrospectively reviewed. We compared the hospital stay, perioperative morbidity, and rate of surgical conversion to open cholecystectomy according to the relief of jaundice before surgery. These enrolled patients were divided into two groups: relief of jaundice before surgery (group 1, *n* = 125) or not (group 2, *n* = 156). Results: The initial total bilirubin level was higher in group 1; however, there were no significant differences in the other baseline characteristics including age, sex, American Society of Anesthesiologists score, previous surgical history, white blood cell count, C-reactive protein, and operative time between the two groups. There was also no significant difference in postoperative hospital stay between the two groups (4.5 ± 3.3 vs. 5.5 ± 5.6 days, *p* = 0.087). However, after ERCP, the waiting time until LC was significantly longer in group 1 (5.0 ± 4.9 vs. 3.5 ± 2.4 days, *p* < 0.001). There were no statistical differences in the conversion rate (3.2% vs. 3.8%, *p* = 0.518) or perioperative morbidity (4.0% vs. 5.8%, *p* = 0.348), either. Conclusions: LC would not be delayed until the relief of jaundice after ERCP since there were no significant differences in perioperative morbidity or surgical conversion rate to open cholecystectomy. Early LC after ERCP may be feasible and safe in patients with cholangitis and cholecystolithiasis.

## 1. Introduction

Previous studies reported that 4–15% of patients with cholecystolithiasis also have coexisting choledocholithiasis [1,2,3,4,5,6]. According to a recent systematic review, when cholecystolithiasis and choledocholithiasis are diagnosed at the same time, laparoscopic cholecystectomy (LC) with simultaneous intraoperative cholangiography and common bile duct (CBD) exploration are recommended since they have a high technical success rate and a short hospital stay [7]. However, LC with simultaneous CBD exploration can only be implemented if the necessary surgical expertise is available [8] and is related to a higher postoperative bile leakage rate [9]. Therefore, endoscopic retrograde cholangiopancreatography (ERCP) followed by LC remains the mainstay for managing coexisting gallbladder (GB) and CBD stones [10,11,12]. In addition, inpatient ERCP in conjugation with cholecystectomy is widely accepted because it is the most efficient treatment algorithm which shortens the length of hospital stay [13].

However, despite the widespread conduct of ERCP prior to LC, the optimal timing for cholecystectomy after acute cholangitis remains unclear [14]. The lack of consensus is also reflected in variations in several reports from different countries; after ERCP, LC was conducted within 24–72 h in some studies, [15,16] whereas elsewhere, LC was conducted later than this [17,18,19]. It is believed that the conduct of early planned LC after ERCP can prevent recurrent biliary complications and reduce operation-related morbidity rates and the hospital stay [20]. However, the clinical reason supporting delayed LC is to allow adequate time for inflammatory changes after the index admission to resolve, and such a delay has been advocated for decades [21]. In addition, ERCP itself can induce inflammation of the hepatoduodenal ligament which makes it difficult to dissect the Calot’s triangle, hence increasing the rate of conversion to open cholecystectomy [22].

Recent studies have advocated early cholecystectomy after ERCP, and the latest randomized controlled trial comparing single-stage ERCP plus LC and two-stage ERCP followed by LC six-to-eight weeks later revealed that single-stage ERCP plus LC was safe and feasible with advantages of cost, shorter hospital stay, and absence of the risk of recurrent episodes of acute cholecystitis, which can occur with delayed cholecystectomy [23]. However, in this study, although the preoperative levels of serum total bilirubin were significantly different between the study groups, the effect of jaundice was not evaluated. Obstructive jaundice with CBD stones and acute cholangitis are related to malabsorption of nutrients, impaired immune response, and disruption of the intestinal mucosal barrier, which could affect the perioperative morbidity [24].

With such a background, this study aimed to determine the relationship between the relief from jaundice after ERCP and subsequent postoperative outcomes in patients undergoing LC for coexisting cholecystolithiasis. Although previous studies only focused on the time interval, in this study, we assessed the optimal timing of LC with a focus on the improvement of jaundice before surgery.

## 2. Methods

### 2.1. Study Population

**Figure 1:**
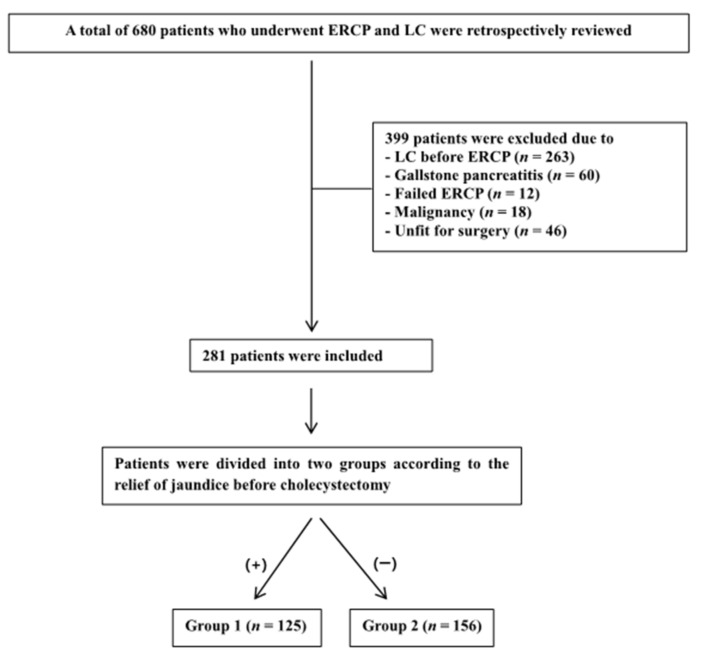
Flow diagram of the study population.

This retrospective study focused on patients who presented with concomitant GB and CBD stones. Medical records of all the patients admitted for ERCP and LC from January 2010 to December 2018 to a single tertiary center were reviewed. The tentative diagnosis of GB and CBD stones was made based on symptoms and signs, abnormal results of liver function tests and diagnostic imaging studies including abdominal ultrasound and computed tomography scans. For cases with ambiguous findings for gallstones, magnetic resonance cholangiopancreatography or endoscopic ultrasonography was conducted. A total of 281 patients with a diagnosis of acute cholangitis as defined by the Tokyo guidelines [25,26] and subsequent confirmed clearance of choledocholithiasis by ERCP were included for analysis. Patients with acute cholecystitis, biliary pancreatitis, failed ERCP, other malignancies including GB cancer, or who were unfit for surgery (e.g., had a coagulopathy or severe complications after ERCP) were excluded from this study (Figure 1). The patients’ age; gender; American Society of Anesthesiologists physical status; white blood cell count; total serum bilirubin, alkaline phosphatase, alanine aminotransferase, aspartate aminotransferase, gamma glutamyl transpeptidase levels; time interval between ERCP and LC; surgical conversion rate to open cholecystectomy; postoperative and total hospitalization period; operation time and intra- and postoperative complications were collected. The length of the postoperative hospitalization period was defined as the number of days between cholecystectomy and the discharge date. The total hospital stay was defined as the period from initial admission to discharge. The study population was divided into two groups, those who obtained relief from jaundice preoperatively (group 1) and those who did not (group 2). The relief from jaundice was defined as a decrement of serum total bilirubin of more than two thirds of the initial level before cholecystectomy [27]. All the operations were conducted using the standard four- or three-port technique. Our primary outcomes were lengths of the postoperative hospital stay and total hospital stay, whereas the secondary outcomes included the conversion rate to open cholecystectomy, the operative time, and the perioperative morbidity rate. Perioperative morbidity included major hemorrhage, bile leakage, surgical infection and perforation of the GB. Major hemorrhage was defined as postoperative bleeding that required additional intervention such as embolization or reoperation. Similarly, the definition of postoperative bile leakage was confined to indicate persistent leakage of bile from the biliary tree that needed re-ERCP or percutaneous transhepatic biliary drainage. Furthermore, the incidence rates of superficial surgical site infections (SSIs) and organ space infections (OSIs) within 30 days of surgery were investigated. Perforation of the GB included traumatic injury that occurred during the operation because of traction or electrocautery dissection of GB.

Additional analysis to identify the variables affecting the rate of improvement of jaundice was conducted. The diameter of the CBD was based on the most dilated part on the ERCP, whereas gallstone size was determined by measuring the diameter of the largest one visible in the image. The definition of ERCP-related complications, including pancreatitis, bleeding, and perforation, followed the American Society for Gastrointestinal Endoscopy guidelines [28].

This study was approved by the relevant institutional review board on March 3, 2015 (IRB No. IB-1503-015), and conformed to the ethical guidelines of the 1975 Declaration of Helsinki (6th revision, 2008). The requirement to procure informed consent of the patients was waived because of the study’s retrospective nature. This study was registered in the Clinical Research Information Service (cris.nih.go.kr, identifier: KCT0005426) and reported in line with the STROCSS criteria [29].

### 2.2. Statistical Analysis

All the continuous variables were expressed as the means ± standard deviations if the variables were normally distributed; otherwise, they were expressed as the medians. The comparison of continuous variables was completed by an independent samples *t*-test, Mann–Whitney test, or Kruskal–Wallis test. Categorical variables were compared using the chi-squared or Fisher’s exact test. The logistic regression analysis was performed to evaluate any clinical factors associated with perioperative morbidity. A two-sided *p*-value of less than 0.05 was considered to be statistically significant. All the statistical analyses were performed using SPSS Statistics version 21.0 for Windows (IBM Corporation, Armonk, NY, USA).

## 3. Results

### 3.1. Baseline Characteristics

A total of 281 patients who underwent LC after ERCP were enrolled, including 125 patients who had undergone ERCP followed by LC after experiencing relief from jaundice before surgery (group 1) and 156 patients who had undergone ERCP followed by LC without experiencing relief from jaundice before surgery (group 2). The baseline characteristics of these patients are described in Table 1. The initial level of total bilirubin was significantly higher in group 1 than in group 2 (5.5 ± 3.8 vs. 3.0 ± 1.5 mg/dL; *p* < 0.001), whereas the preoperative level of total bilirubin was significantly higher in group 2 than in group 1 (1.6 ± 1.0 vs. 1.2 ± 0.9 mg/dL, *p* = 0.002). Moderate or severe cholangitis was more common in group 1 than in group 2 (67.2% vs. 47.4%, *p* = 0.001).

### 3.2. Study Outcomes

The mean time intervals between ERCP and LC were 5.0 ± 4.9 days for group 1 and 3.5 ± 2.4 days for group 2, with a significant difference present between the two groups (*p* = 0.001). Endoscopic nasobiliary drainage (ENBD) was usually placed after ERCP to check for residual stones before cholecystectomy (56.0% in group 1, 51.3% in group 2).

LC was technically successful in 271 (96.4%) patients, whereas the remaining 10 patients required conversion to open cholecystectomy. The mean operative time was not significantly different between the two groups either (group 1 vs. group 2: 64.9 ± 31.4 vs. 72.1 ± 34.8 min; *p* = 0.072). Conversion to open cholecystectomy was conducted in four cases in group 1 and six cases in group 2. Nevertheless, there was no significant difference in the conversion rate between the two groups (3.2% vs. 3.8%; *p* = 0.518). All the patients in both groups underwent LC without experiencing postoperative mortality.

The perioperative morbidity rate including major hemorrhage, bile leakage, perforation, and infection was not significantly different between the two groups (4.0% vs. 5.8%; *p* = 0.348). A total of five cases of postoperative infection were identified, three of which were in group 1 and two of which were in group 2, without a statistically significant difference between the groups. Additionally, upon subdividing the infections, four were ruled to be organ space infections and the other one was a superficial surgical site infection. In logistic regression regarding the perioperative morbidity, the preoperative serum total bilirubin levels were not associated with perioperative morbidities (HR, 1.35, 95% CI, 0.94–1.95, *p* = 0.103). In multivariable analysis including preoperative serum total bilirubin, age, sex, American Society of Anesthesiologists physical status, previous surgical history, and any ERCP-related complications, there were no significant risk factors associated with perioperative morbidity. Details pertaining to the primary and secondary study endpoints from both groups are described in Table 2. The length of hospital stay after the operation was similar between the two groups (4.5 ± 3.3 vs. 5.5 ± 5.6 days; *p* = 0.087).

During the same hospitalization period, additional ERCP was performed in 13 patients. Among these, additional ERCP was performed in six patients (4.2%) in group 1 and in seven patients (5.0%) in group 2, respectively (*p* = 0.747). Among them, seven patients with residual choledocholithiasis on cholangiography with ENBD and one patient with post-ERCP bleeding underwent ERCP before surgery, and the remaining five patients underwent ERCP after surgery due to bile leakage (Table 3).

To discern any confounders that may be correlated with the relief from jaundice, the factors that helped improve serum bilirubin levels after ERCP were also analyzed. Ultimately, however, it was found that the diameter of the CBD, the features of the gallstones and ERCP-related factors did not influence the decrement of serum bilirubin levels significantly (Table 4).

## 4. Discussion

Up to 19% of patients undergoing LC have CBD stones that may require CBD exploration, [30] and ERCP is replacing intraoperative cholangiography with CBD exploration in these patients [1,2,3,4,5,6,9]. According to this trend, studies on the optimal surgical timing after ERCP were required in hospitalized patients with biliary complications [13]. Earlier LC after ERCP or even single-stage ERCP and LC seemed to be feasible and efficacious in previous studies; [20,23] however, most of these studies only focused on the time interval between ERCP and LC without considering whether jaundice improved before LC. Therefore, in this study, we assessed the safety of early LC after ERCP with a greater focus on the relief from jaundice than on the time interval between ERCP and LC. The primary outcomes in our study were the lengths of postoperative hospital stay and total hospital stay. The secondary outcomes included the conversion rate to open surgery, the operative time, and the postoperative morbidity rate. Ultimately, the length of hospitalization after LC was similar between the two groups. Similarly, the conversion rate to open cholecystectomy was 3.2% in group 1 and 3.8% in group 2, stressing no significant difference existed between the two groups. The rate of perioperative morbidity, which included hemorrhage, bile leakage, and perforation, also showed no significant difference between the two groups (4.0% in group 1 vs. 5.8% in group 2; *p* = 0.348). Our results suggested that earlier LC after ERCP regardless of the relief from jaundice was feasible—that is, the procedure could be conducted without leading to significant adverse events. Therefore, LC may not be delayed after the relief from jaundice and could be conducted during the index admission.

Several previous studies have advocated for early LC, claiming that earlier surgery can lead to a significant reduction in the total length of hospital stay and medical costs, consistent with our results [31,32,33,34,35] Moreover, the recently published meta-analyses and a prospective study regarding gallstone pancreatitis also recommended early cholecystectomy [36,37]. These studies stated that a major disadvantage of delayed LC is that patients could be lost before surgery while carrying the risk of developing gallstone-related complications that require endoscopic reintervention, emergency cholecystectomy, or both. In our study, only five (1.7%) patients were readmitted to hospital for ERCP due to recurrent cholangitis after cholecystectomy, although it was impossible to identify whether cholangitis was caused by residual stones or newly passed stones during LC. Previous studies suggested that the incidence of biliary complications requiring endoscopic reintervention is as high as 20% in patients who have undergone delayed LC [20,38]. For patients who experienced these recurrent events, the rates of perioperative morbidity and conversion to open cholecystectomy and the length of postoperative hospitalization are sharply increased [20]. These results suggest that early LC after ERCP in patients with concomitant choledocholithiasis and cholecystolithiasis is feasible and safe, without palliation of cholestasis. Therefore, LC may not have to be postponed until the relief from jaundice is obtained. Of note, although the improvement of jaundice did not affect the surgical outcome and prognosis in this study, clinicians tend to prefer operating on patients showing good liver function, with normal bilirubin levels in practice. Therefore, further analysis was conducted on the factors affecting the rate of jaundice improvement, but no meaningful factors were found.

This study has some limitations. First, since our study was retrospective in nature, several data related to the operation were missing. Additional data about intraoperative findings including adhesion grades, periportal inflammation, and fibrosis could be helpful in determining the optimal time of LC. A randomized controlled trial with a larger number of patients is required to further assess our results. In addition, the treatment policy for the time interval between ERCP and LC may vary according to the countries’ medical insurance systems. Although prompt LC following ERCP can prevent biliary complications including recurrent cholangitis or cholecystitis, LC after ERCP during the same admission is not available in some countries because of their insurance systems. Since the Korean medical insurance guarantees coverage of most costs of treatment for all citizens, both choledocholithiasis and cholecystolithiasis are usually treated in the same admission. Therefore, our findings may not be equally applicable to all countries. Finally, since the definition of relief from jaundice is also different in each of the previous studies, further research on the standard criteria for such is essential.

In conclusion, LC may not have to be delayed because there are no significant differences in rates of perioperative morbidity and open cholecystectomy and the length of hospitalization. Thus, early LC after ERCP without relief from jaundice may be feasible in patients with coexisting cholecystolithiasis and choledocholithiasis with cholangitis.

## Figures and Tables

**Table 1:** Initial baseline characteristics of the enrolled patients.

Variables (Mean ± SD)	Group 1 (*n* = 125)	Group 2 (*n* = 156)	*p*
Age (years)	59.1 ± 17.2	59.8 ± 16.9	0.730
Sex (male/female)	73/52	103/53	0.215
ASA (*n*, %) Category 1 Category 2 Category 3	- 38 (30.4%) 74 (59.2%) 13 (10.4%)	- 58 (37.2%) 83 (53.2%) 15 (9.6%)	0.309
Previous surgical history (*n*, %)	21 (16.8%)	29 (18.6%)	0.755
WBC (K/mm^3^)	9.0 ± 3.0	8.9 ± 4.1	0.840
CRP (mg/dL)	2.4 ± 3.8	3.2 ± 4.2	0.214
Creatinine (mg/dL)	0.8 ± 0.2	0.8 ± 0.2	0.990
Initial total bilirubin (mg/dL)	5.5 ± 3.8	3.0 ± 1.5	<0.001
Preoperative total bilirubin (mg/dL)	1.2 ± 0.9	1.6 ± 1.0	0.002
ALT (units/L)	382.9 ± 260.8	288.1 ± 249.7	0.002
AST (units/L)	363.8 ± 312.8	301.9 ± 357.6	0.129
ALP (units/L)	222.9 ± 171.1	197.4 ± 127.2	0.154
r-GT (units/L)	521.4 ± 326.9	453.3 ± 321.9	0.082
Severity of cholangitis (*n*, %) Mild Moderate oo severe	- 41 (32.8%) 84 (67.2%)	- 82 (52.6%) 74 (47.4%)	0.001
Interval between ERCP and LC (days)	5.0 ± 4.9	3.5 ± 2.4	0.001
Operative time (min)	64.9 ± 31.4	72.1 ± 34.8	0.072

ASA: American Society of Anesthesiologists physical status; WBC: white blood cell; CRP: C-reactive protein; ALT: alanine aminotransferase; AST: aspartate aminotransferase; ALP: alkaline phosphatase; r-GT: gamma-glutamyl transpeptidase; ERCP: endoscopic retrograde cholangiopancreatography; LC: laparoscopic cholecystectomy.

**Table 2:** Comparison of primary and secondary endpoints between the two groups.

Variables	Group 1 (*n* = 125)	Group 2 (*n* = 156)	*p*
Primary endpoints			
Postoperative hospital stay (days, mean ± SD)	4.5 ± 3.3	5.5 ± 5.6	0.087
Total hospital stay (days, mean ± SD)	11.2 ± 5.0	11.1 ± 6.8	0.874
Secondary endpoints (*n*, %)			
Conversion rate to open cholecystectomy	4 (3.2%)	6 (3.8%)	0.518
Perioperative morbidity	5 (4.0%)	9 (5.8%)	0.348
Major hemorrhage	0 (0.0%)	1 (0.6%)	0.555
Bile leakage	2 (1.6%)	3 (1.9%)	0.603
Infection	3 (2.4%)	2 (1.3%)	0.397
Perforation	0 (0.0%)	3 (1.9%)	0.170

**Table 3:** The details of the patients who underwent additional ERCP.

Patient ID	Sex	Age (Years)	Group	Number of the Additional ERCP	Timing of the Additional ERCP	Reason for the Additional ERCP
1	M	85	2	1	Preoperative	Residual stone confirmed by ENBD cholangiography
2	M	68	2	1	Preoperative	Residual stone confirmed by ENBD cholangiography
3	M	76	2	1	Preoperative	Residual stone confirmed by ENBD cholangiography
4	M	76	2	1	Preoperative	Residual stone confirmed by ENBD cholangiography
5	F	46	2	1	Preoperative	Residual stone confirmed by ENBD cholangiography
6	M	61	1	2	Preoperative	Residual stone confirmed by ENBD cholangiography
7	M	48	1	1	Preoperative	Residual stone confirmed by ENBD cholangiography
8	M	57	1	1	Preoperative	Post-ERCP bleeding
9	M	58	2	1	Postoperative	Bile leakage after cholecystectomy
10	F	74	2	1	Postoperative	Bile leakage after cholecystectomy
11	F	72	2	1	Postoperative	Bile leakage after cholecystectomy
12	F	49	1	1	Postoperative	Bile leakage after cholecystectomy
13	F	36	1	1	Postoperative	Bile leakage after cholecystectomy
14	F	33	2	1	After discharge	Recurrent cholangitis during the follow-up period
15	F	75	2	1	After discharge	Recurrent cholangitis during the follow-up period
16	M	82	2	1	After discharge	Recurrent cholangitis during the follow-up period
17	M	76	2	1	After discharge	Recurrent cholangitis during the follow-up period
18	M	76	1	1	After discharge	Recurrent cholangitis during the follow-up period

**Table 4:** Comparison of the procedure-related factors between the two groups.

Variables (Mean ± SD)	Group 1 (*n* = 125)	Group 2 (*n* = 156)	*p*
Diameter of the common bile duct (mm)	11.0 ± 3.8	11.0 ± 3.5	0.852
Size of stone (mm)	5.8 ± 3.6	5.9 ± 3.6	0.911
Number of stones	1.4 ± 0.9	1.4 ± 0.8	0.458
Nature of stones Brown pigmentation Black pigmentation Cholesterol	- 77 (61.6%) 30 (24.0%) 18 (14.4%)	- 103 (66.0%) 32 (20.5%) 21 (13.5%)	0.539
Complete removal of stones Yes No	- 117 (93.6%) 8 (6.4%)	- 143 (91.7%) 13 (8.3%)	0.873
Method of papillary dilation EST EPBD EST + EPBD	- 99 (79.2%) 9 (7.2%) 17 (13.6%)	- 103 (66.0%) 32 (20.5%) 21 (13.5%)	0.106
Placement of a prosthesis for biliary drainage None ERBD ENBD	- 46 (36.8%) 9 (7.2%) 70 (56.0%)	- 69 (44.2%) 7 (4.5%) 80 (51.3%)	0.294
Post-ERCP related complications None Pancreatitis	- 106 (84.8%) 18 (14.4%)	- 138 (88.5%) 17 (10.9%)	0.385
Bleeding	1 (0.8%)	1 (0.6%)	
Perforation	0	0	

EST: endoscopic sphincterotomy; EPBD: endoscopic papillary balloon dilation; ERBD: endoscopic retrograde biliary drainage; ENBD: endoscopic nasobiliary drainage.

## Data Availability

The data presented in this study are available upon request from the corresponding author.
